# Addressing the Insufficient Availability of EPA and DHA to Meet Current and Future Nutritional Demands

**DOI:** 10.3390/nu13082855

**Published:** 2021-08-20

**Authors:** Sunil K. Panchal, Lindsay Brown

**Affiliations:** 1 School of Science, Western Sydney University, Richmond, NSW 2753, Australia; S.Panchal@westernsydney.edu.au; 2 School of Pharmacy and Medical Science, Griffith University, Southport, QLD 4222, Australia

Tocher and colleagues [1] have summarised the widening gap between the supply of EPA and DHA and the amounts required to achieve a healthy dietary intake [2,3,4]. A large global study identified the mean individual consumption of long-chain omega-3 fatty acids (EPA and DHA) in adults to be 163 mg/day in 2010 [5]. The dietary recommendations for EPA and DHA range from 250 to 1000 mg/day for adults [6], but three billion people have an intake of <100 mg/day [5]. Dosages of 500 mg/person/day would require ~1.27 million tonnes/year of long-chain omega 3 fatty acids for a population of approximately seven billion in 2019, indicating a shortfall of 0.4–1.0 million tonnes/year [1]. Thus, identifying solutions to provide adequate supplies of these fatty acids is a current and future requirement.

Early human diets included a balanced intake of omega-3 and omega-6 fatty acids for nutrition. However, this balance has been changed in the typical Western diet with the ratio of omega-6 to omega-3 fatty acids now being 20:1 or higher [7]. This increase in omega-6 and decrease in omega-3 fatty acids has been proposed as a major cause of increased chronic disease [7]. Thus, the reasons for increasing EPA/DHA in the diet now include health improvement in chronic disease states [8]. With both an increasing population and an increasing prevalence of chronic diseases as the population ages, future demands for EPA/DHA will further increase. We have previously reviewed the beneficial effects and mechanisms of action of these long-chain omega-3 fatty acids [9]. With increasing demand for these beneficial fatty acids, supply has become a major challenge and has an important impact on health and commercial outcomes.

Traditionally, the major source of EPA/DHA in the human diet has been the harvesting of fish from natural marine and freshwater environments. However, these capture fisheries as a source are at or beyond their sustainable limits [1], and global aquaculture production has increased by more than five times from 1990 to 2018 [10]. Aquaculture fisheries need sources of EPA/DHA as fish do not produce these compounds on their own. However, the use of waste fish products as fishmeal for aquaculture is finite but increases the risk of pollution with increased ammonia, phosphate and protein [11]. Furthermore, fishmeal requires the use of antioxidants such as ethoxyquin for stabilisation against lipid peroxidation; the toxicity of this compound and its oxidation products detected in farm animals need better definition [12]. Avoiding use of fishmeal would then seem the safest option. Increasing EPA/DHA production without using fishmeal includes applying 19th/20th century approaches to find new zooplankton sources of EPA/DHA for aquaculture [1].

An alternate source of EPA/DHA for farmed fish as discussed by Tocher and colleagues is microalgae, as these organisms convert carbon dioxide and sunlight into complex molecules such as omega-3 fatty acids, carotenoids, proteins and carbohydrates [1]. The obvious shortcut is then to include microalgal EPA/DHA directly in human foods and supplements to bypass the production of fish by aquaculture as a source of EPA/DHA.

Microalgae not only have the advantage of providing omega-3 fatty acids but can also tackle the increased water pollution during increased production of fish in aquaculture by recycling the wastewater from aquaculture to cultivate microalgae [11]. Thus, microalgae can complete the circular economy where fish are grown with microalgae as the source of EPA/DHA while producing pollutants that can be bioremediated by growing microalgae which can then be fed to the fish again. This option is not available when fishmeal is used as the source of EPA/DHA.

Omega-3 fatty acids, such as α-linolenic acid, are produced by plants, including flax (linseed), but conversion to EPA and DHA in humans is poor. However, introduction of a microalgal/yeast transgenic pathway to canola produced similar concentrations of DHA as in fish oil [13]. As a dietary supplement, seed oil from another transgenic brassica, *Camelina sativa*, containing EPA and DHA was as effective as fish oil in increasing EPA and DHA concentrations in humans [14]. Possible disadvantages of these crops include the use of decreasing resources such as valuable agricultural land and water for irrigation; these issues are markedly diminished with microalgal production of EPA/DHA.

Microalgae have been defined as a biorefinery because of the range of compounds that they produce [15]. In addition to providing sustainable food sources for monogastric animals and humans [16,17], microalgae produce additional compounds with possible biomedical applications, such as carotenoids, chlorophyll and prebiotics. Furthermore, microalgae are feasible sources for biofuels [18] and for bioremediation of urban wastewater [19] or water contaminated with heavy metals [20] or pharmaceuticals and primary care products [21]. Therefore, *Nannochloropsis* grown for other uses could be diverted for the development of nutraceuticals and pharmaceuticals at minimal additional cost. These options are not available when EPA and DHA are supplied from fishmeal.

While microalgae and plants can replace fishmeal as alternate omega-3 fatty acid sources for aquaculture, protein sources for growing fish are also important to consider. Microalgae also provide essential proteins for aquaculture, thus improving the quality of fish grown using microalgae. Soybean meal has been considered as an alternate source for protein and showed no adverse effects on optimal growth and feed utilisation [22]. Furthermore, using soybean meal for formulation of diets for growing European sea bass showed appropriate nutritional status and no evidence of soy-induced enteritis. However, soybean requires significant resources, especially agricultural land and water.

In summary, addressing the current gap between the supply and demand of EPA/DHA has become an important issue in the nutraceutical field. Sufficient supply of these long-chain omega-3 fatty acids will help achieve a healthy dietary intake of these important nutrients. Tocher and colleagues summarised this gap between supply and demand of EPA/DHA, indicating that we not only have a shortage in the supply of EPA/DHA for producing more EPA/DHA, but we also have a shortage of intake in current consumers [1]. Furthermore, they discussed the role of aquaculture in providing these nutrients, potential novel sources and ways for their effective use [1]. With increasing demands for EPA/DHA to address increasing chronic diseases, it is necessary to identify solutions for providing additional supplies of these omega-3 fatty acids to improve current and future health worldwide. Microalgae could be the most effective solution.
